# A new feasible technique of mesh-reinforced pancreatojejunostomy and pancreatogastrostomy: retrospective analysis of 61 cases

**DOI:** 10.1186/1477-7819-10-114

**Published:** 2012-06-22

**Authors:** Wang Xianfa, Xin Ying, Pan Junhai, Zhang Nengyun, Zhou Wei

**Affiliations:** 1 Department of General Surgery, Sir Run Run Shaw Hospital, Medical School, Zhejiang University, Institute of Micro-invasive Surgery of Zhejiang University, No.3, Qin Chun Road, Hangzhou, China

**Keywords:** Pancreatogastrostomy, Pancreatojejunostomy, Mesh

## Abstract

**Background:**

Pancreatic leak was the major concern after pancreatoduodenectomy.

**Methods:**

A total of 61 patients who underwent mesh-reinforced pancreatojejunostomy or pancreatogastrostomy from August 2005 to November 2011 were retrospectively analyzed.

**Results:**

The mean anastomosis time of mesh-reinforced pancreatojejunostomy was 25 minutes ranging from 22 to 35 minutes. In mesh-reinforced pancreatogastrostomy, the mean anastomosis time ranged from 20 to38 minutes with an average of 30 minutes. Blood loss was 200 to 4,000 ml with an average of 710 ml in all patients. There was one case of pancreatic leak of Class A, three cases of pancreatic leak of Class B, one case of pancreatic leak of Class C, one case of choledochojejunostomy leakage, one case of gastrojejunostomy leakage, and three cases of abdominal bleeding.

**Conclusion:**

As a new technique, mesh-reinforced pancreatojejunostomy and pancreatogastrostomy might be a safe and feasible procedure to prevent postoperative pancreatic leak.

**Trial registration:**

This research is waivered from trial registration because it was a retrospective analysis of medical records.

## Background

Pancreatic leak was still the major concern after pancreatoduodenectomy. Various reconstructions for pancreatic remnant have been explored including end-to-end invaginated pancreaticojejunostomy, duct-to-mucosa pancreaticojejunostomy, binding pancreatogastrostomy or pancreatojejunostomy, and so on. However, surgeons are still striving for a safer and more feasible procedure.

The incidence of pancreatic leak varied in studies and this was unsatisfying. We designed mesh-reinforced reconstruction of pancreatojejunostomy and pancreatogastrostomy. In our institution, 61 patients underwent this new method successfully. We report the preliminary results as follows.

## Methods

From August 2005 to November 2011, 61 (33 males and 28 females) cases of mesh-reinforced pancreatojejunostomy and pancreatogastrostomy were performed and retrospectively analyzed in our institution. The age ranged from 19 to 78 years with an average of 58 years. Among the 61 patients, there were 29 cases of malignancy in lower common bile duct, 13 cases of pancreatic head carcinoma, 9 cases of duodenal papilla carcinoma, 6 cases of cystoadenoma in pancreatic head, 1 case of duodenal papilla adenoma, 1 case of duodenal malignant stroma, 1 case of pancreatic trauma and 1 case of colon carcinoma. In our institution binding pancreaticojejunostomy (end-to-end) was the first choice for reconstruction which was invented by Professor Peng [1]. Six patients had edematous, fragile and enlarged pancreatic remnant so we decided to perform pancreaticogastrostomy.

The study was approved by the Committee of Ethics of Sir Run Run Shaw Hospital of Zhejiang University. All patients signed a written informed consent with the potential surgical risks. The same surgical team was responsible for all procedures in the study.

### Surgical technique

After patients were generally anesthetized, an incision was made using either epigastric reversed ‘L’-shaped incision or roof-like incision below bilateral costal ribs. Abdomen was explored to rule out distal metastasis before pancreatoduodenectomy. Pancreatoduodenectomy was completed as a routine procedure.

**Figure 1:**
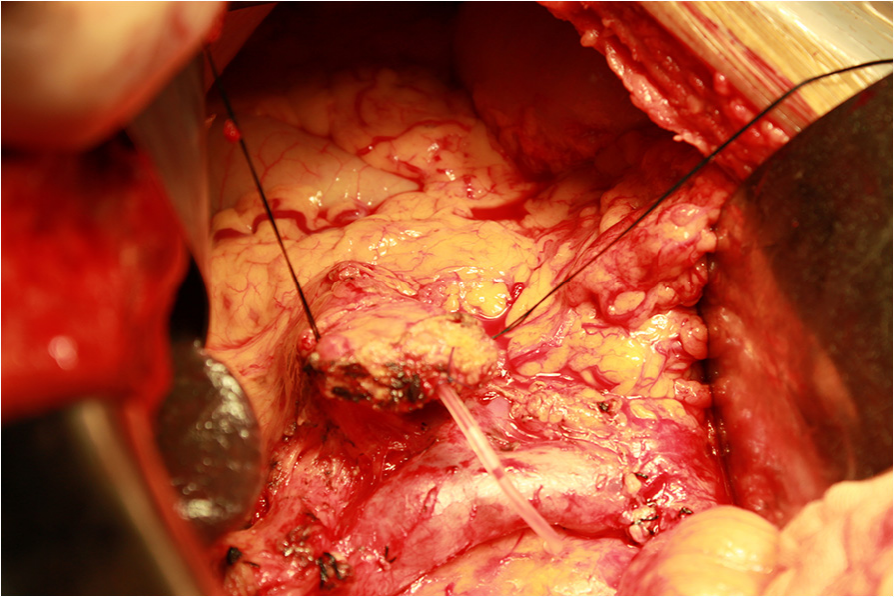
Pancreatic stump was mobilized 2 to 3 cm in length. A stent was inserted into pancreatic duct.

**Figure 2:**
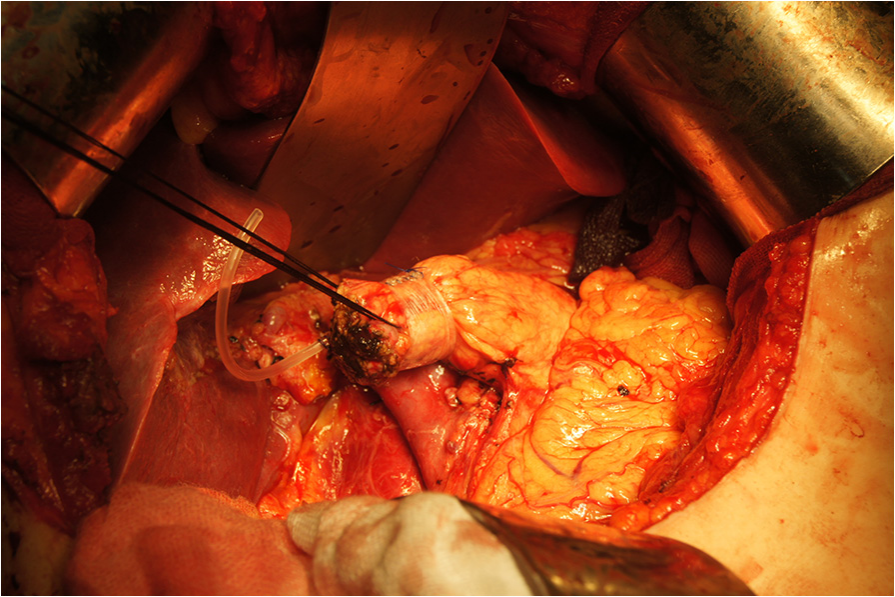
Pancreas was wrapped in one circle using mesh.

After surgical sample removal, the pancreatic remnant was mobilized 2 to 3 cm in length. A stent was inserted into pancreatic duct (Figure 1). Non-absorbable (polypropylene mesh, big pores, Ethicon, New Jersey, USA) or absorbable (Cook, Limerick, Ireland) hernia graft was used for pancreatic remnant reconstruction. Mesh was cut into 1.5 cm widths. The length of mesh should match with the circumferential length of pancreas. Pancreas was wrapped in one circle using mesh, which was sutured by 3–0 prolene to close ends (Figure 2).

#### End-to-end pancreatojejunostomy

**Figure 3:**
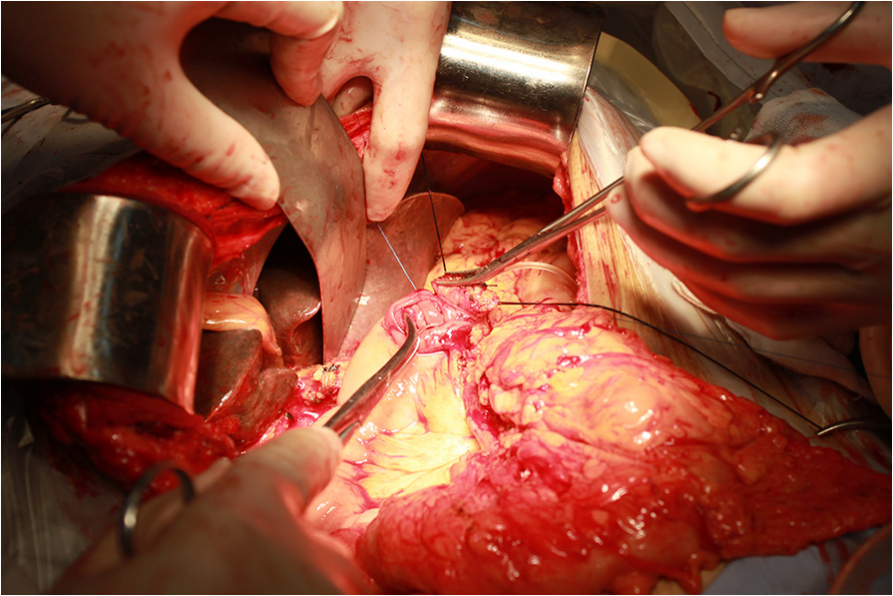
Posterior part of bowel loop was fixed to left edge of mesh in posterior pancreatic stump using 4–0 prolene in continuous suture.

**Figure 4:**
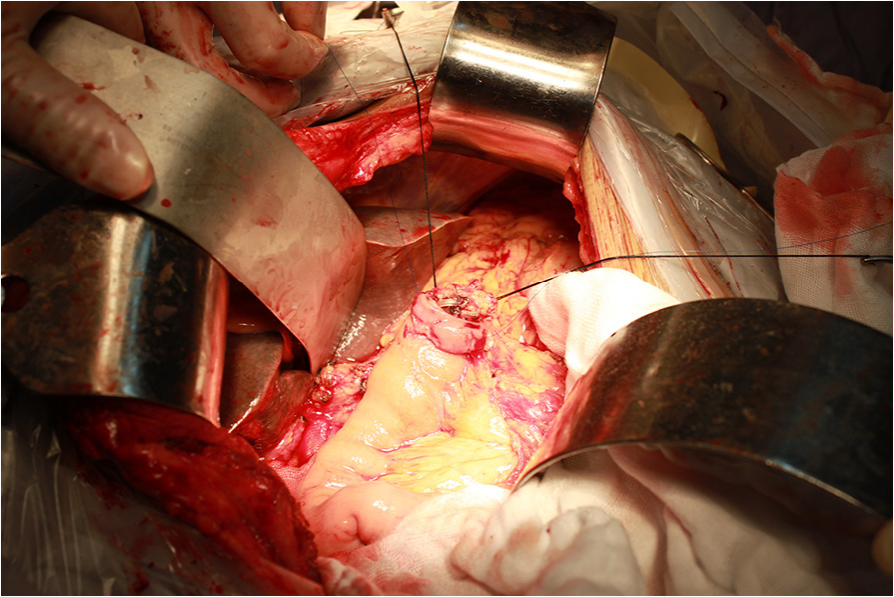
Anterior part of jejuna loop was fixed to left edge of mesh in anterior pancreatic stump too.

**Figure 5:**
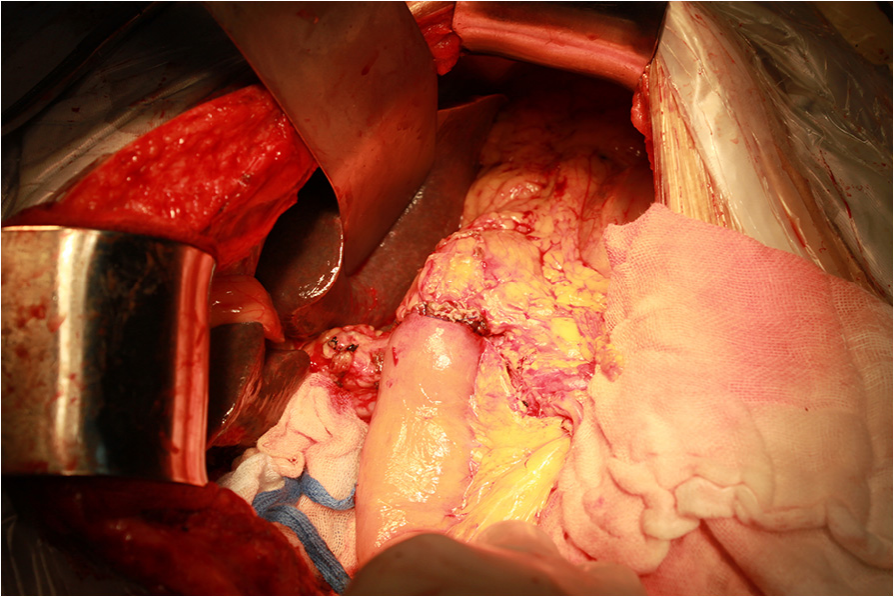
Mesh was wrapped by bowel loop completely after prolene was fastened.

Jejunal loop was lifted upward behind colon. The posterior part of jejunal stump was sutured to left edge of mesh in pancreas using continuous 4–0 prolene stitches (Figure 3). The anterior part of jejuna loop was fixed to left edge of mesh in pancreatic stump too thereafter (Figure 4). Mesh should be wrapped by bowel loop completely after prolene was fastened (Figure 5). The leak test was performed after anastomosis was completed (50 ml of methylene blue in syringe was injected into bowel to reach the pressure of 25 cmH_2_O).

#### Pancreatogastrostomy

**Figure 6:**
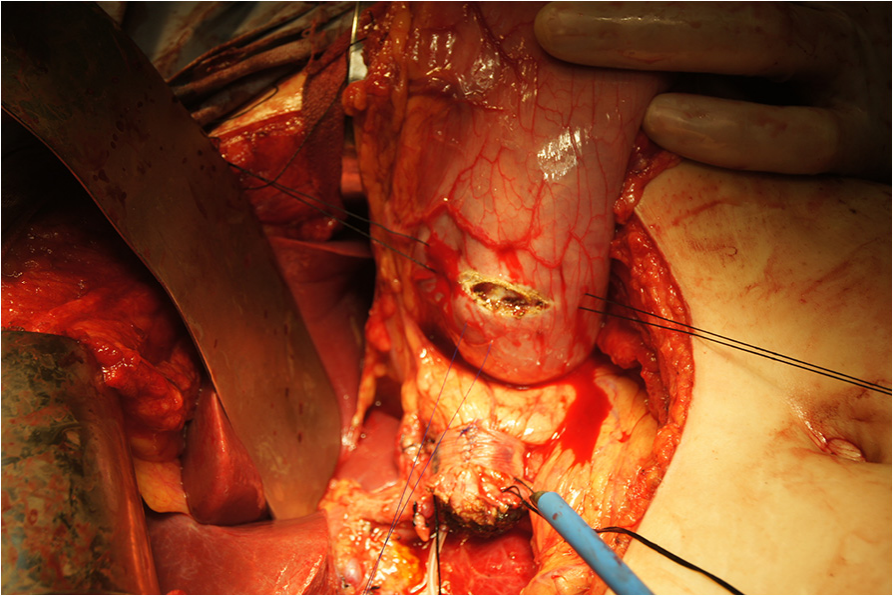
A patch of posterior wall of stomach was excised.

**Figure 7:**
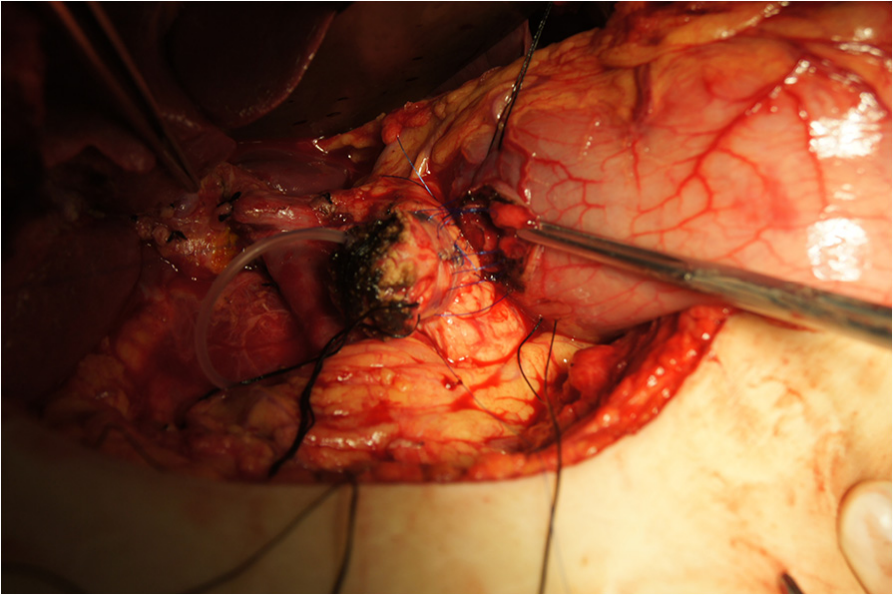
Inner purse-string suture was pre-placed between stomach and left edge of mesh in pancreas using 4–0 prolene.

**Figure 8:**
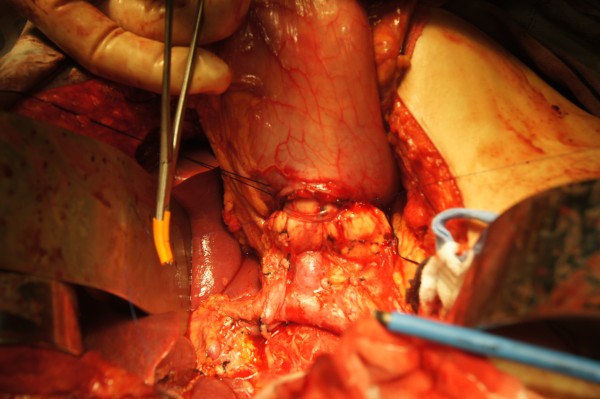
Outer purse-string suture was made between stomach and right edge of mesh in pancreas thereafter.

**Figure 9:**
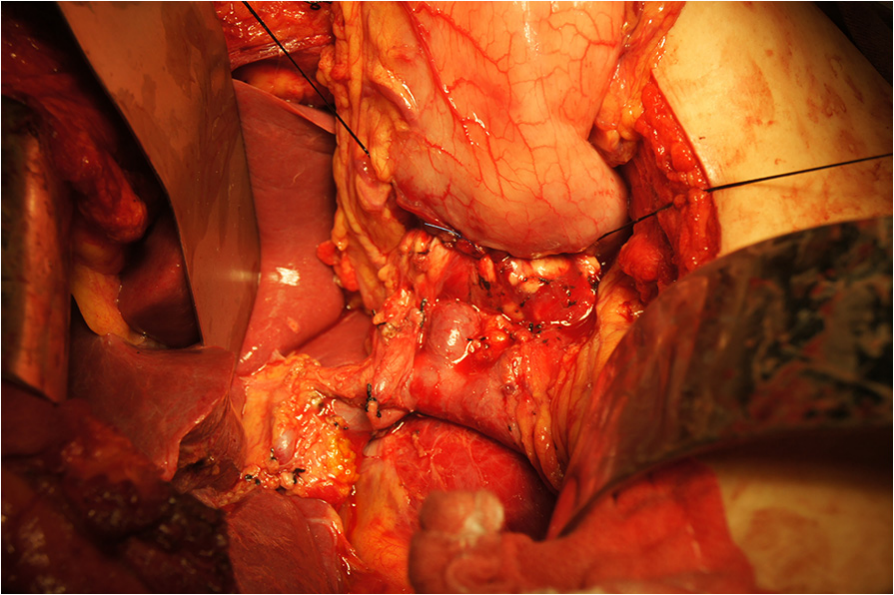
Pancreatic stump was intussuscepted into gastric cavity and pancreatogastrostomy was completed.

Firstly, a patch of posterior wall of stomach was excised (Figure 6) and an inner purse-string suture was pre-placed between stomach and left edge of mesh in pancreas using 4–0 prolene. After the prolene suture was fastened, the pancreatic stump was invaginated into the gastric cavity (Figure 7). Secondly, an outer purse-string suture was made between stomach and right edge of mesh in pancreas thereafter (Figure 8). Finally, pancreatogastrostomy was completed (Figure 9). A leak test was performed routinely.

Surgical time, anastomosis time, and blood loss was recorded. Postoperative complication was observed including abdominal infection and bleeding. Drain amylase levels were analyzed on day 3, 7, and 10 after operation respectively.

#### Definition of pancreatic leak

Pancreatic leak was defined as a drain volume of more than 50 ml for more than 10 days and the amylase level was 3 times more than serum amylase level [1]. Pancreatic leak was categorized into three classes which were Class A, B and C [2]. Class A was defined as good general condition, negative ultrasonography or computed tomography (CT) finding, no need of drainage longer than 3 weeks, no reoperation, no motality relating to pancreatic leak, no infection or septicemia and no readmission. Class B was defined as ordinary general condition, negative or positive ultrasonography or CT finding, patients suffered from infection, readmission may be required and drainage longer than 3 weeks was common. However, no reoperation, no septicemia and no motality relating to pancreatic leak was found in group of Class B. Class C was defined as bad general condition, positive ultrasonography or CT finding, drainage longer than 3 weeks was needed, and reoperation and readmission was required. Patients of Class C may suffer from infection or septicemia. There was motality relating to pancreatic leak in group of Class C.

### Statistical analysis

Data was entered and analyzed using descriptive analysis with SPSS 11.0 (SPSS Inc., Illinois, USA).

## Results

The average surgical time was 6.9 hours for all patients. The average of blood loss volume was 710 ml ranging from 200 to 4,000 ml. A total of 55 patients underwent pancreatojejunostomy and 6 patients underwent pancreatogastrostomy in the study. A total of 53 patients were followed after surgery. The average follow-up time was 14 months ranging from 2 to 25 months. In our study 8 cases lost of contact during follow-up. 5 cases died during follow-up. The dead cases were all pancreatic head carcinoma and they died 1.0 to 1.5 years after surgery.

### Mesh-reinforced pancreatojejunostomy

The anastomosis time of mesh-reinforced pancreatojejunostomy ranged from 22 to 35 minutes with an average of 25 minutes. No anastomosis leakage was found during leak test with pressure of 25 cmH_2_O. The drain volumes in 8 cases were more than 50 ml per day. The amylase levels of 10 cases were 3 times more than serum amylase levels (Table 1).

**Table 1:** Pancreatic leak on day 3, 7 and 10 after surgery

**Postoperative Time (day)**	**Drainage volume per day (cases*n*)**		**Amylase level of drainage tube (cases*n*)**	
	**<50 ml**	**>50 ml**	**<3 × serum amylase level**	**>3 × serum amylase level**
**3**	35	26	33	28
**7**	44	17	45	16
**10**	53	8	51	10

There was one case of pancreatic leak of Class A, 3 cases of pancreatic leak of Class B, one case of pancreatic leak of Class C, one case of choledochojejunostomy leakage, one case of gastrojejunostomy leakage, and three cases of abdominal bleeding. In our study two cases were diagnosed as pancreatic head cancer with acute pancreatitis before operation. No pancreatic leak occurred in these two cases after operation. However one case had choledochojejunostomy leakage with gastrojejunostomy leakage. A total of 18 cases (18/61, 29.5%) suffered from postoperative fever higher than 38.5°C, among which 6 cases (9.8%) had abdominal fluid collection or infection. All patients recovered well after conservative treatment except that one case of pancreatic leak of Class C had a reoperation.

### Mesh-reinforced pancreatogastrostomy

In 6 cases of mesh-reinforced pancreatogastrostomy, the mean anastomosis time ranged from 20 to 38 minutes with an average of 30 minutes. A leak test was negative in all patients. No postoperative leakage was observed.

## Discussion

Pancreatic leak is the major concern after pancreatoduodenectomy. Surgeons are still striving for a safer and better procedure to avoid pancreatic leak [1,3,4]. Literature reported that the incidence of pancreatic leak was 11% in end-to-end invaginated pancreatojejunostomy [5] which was widely used. We designed a new method, mesh-reinforced anastomosis, in 2005, and 61patients underwent this new procedure successfully in our study.

The advantages of mesh-reinforced anastomosis [6,7] included as follows. First, mesh provided a safe anchor site for the suture which was especially suitable for the soft and fragile pancreatic texture to avoid postoperative leakage and bleeding. Second, the shape change of the pancreas after mesh reinforcing made the pancreatic stump easily wrapped by bowel loop. Third, suture tightening between posterior wall of bowel loop and left edge of mesh facilitated pancreas invaginating into bowel loop. Fourth, mesh compression to pancreatic tissue decreased the likelihood of pancreatic leak and bleeding. Fifth, mesh stimulated growth of fibroblast and promoted adhesion between pancreas and bowel pouch.

This study included 55 cases of pancreatojejunostomy and 6 cases of gastrojejunostomy. The average time for pancreatojejunostomy was 25 minutes. The leak test result was all negative during operation with pressure of 25 cmH_2_O. Three cases demonstrated that the new method was practical and suitable especially for tough cases. Cases 1 and 2 were patients of pancreatic head cancer with acute pancreatitis. The pancreas was found to be fragile and severely edematous during surgery and easily torn during suturing. Case 3 was a patient of pancreatic trauma with bleeding 1 week after end-to-end invaginated pancreatojejunostomy. During reoperation it was found that two-thirds of the circumference of pancreatojejunostomy was torn off. These three cases underwent mesh-reinforced pancreatojejunostomy successfully and had postoperative bleeding which was cured conservatively.

We supposed bleeding might be due to patients’ general conditions (two cases of pancreatic head carcinoma with pancreatitis and one case of reoperation), but not trypsin digestion. For absorbable mesh, we did not have a case of reoperation after mesh-reinforcement reconstruction so we did not know about the mesh’s absorption in humans. In an experiment with a dog we found that Biodesign hernia graft took 2 months to be absorbed. We therefore thought it was long enough for the growth of anastomosis. Mesh reinforcement may decrease the incidence of bleeding, especially for challenging cases.

### Pancreatic leak after mesh-reinforced anastomosis

According to Bassi’s pancreatic leak classification [2], there was one case of pancreatic leak of Class C (1.6%), three cases of pancreatic leak of Class B (4.9%) and four cases of pancreatic leak of Class A (6.5%) in the group of mesh-reinforced pancreatojejunostomy. In total, seven cases of Class A and B recovered after non-invasive therapy. The reason for pancreatic leak of Class A and B may relate to minor tearing or needle fissure during suturing. It could be prevented by binding pancreatojejunostomy invented by professor Peng [8,9]. There was one case of pancreatic leak of Class C. The preoperative diagnosis was colon carcinoma involved with hepatic flexure. The clinical manifestation included incomplete bowel obstruction and gastrointestinal bleeding. The drain amylase levels were all normal on day 1, 3, 5, and 7 after surgery. However, on day 11 after surgery the patient was diagnosed as pancreatic leak. Reoperation disclosed leakage from pancreatojejunostomy.

In our study, only one case of pancreatic leak (1.6%) required reoperation. No pancreatic leak was observed in group of mesh-reinforced pancreatogastrostomy. The incidence of pancreatic leak was encouraging.

### Could mesh increase the possibility of abdominal infection?

Infection is the major concern for surgeons when using prolene-mesh. However, data in literatures showed no abdominal abscess was observed in cases using mesh for pancreaticojejunostomy [6,10]. Study in absorbable mesh had a similar result which also demonstrated no abdominal infection occurred [11].

Our data also showed no prolonged abdominal infection due to mesh was observed which may be because mesh was completely wrapped by bowel loop or stomach in the procedure.

However, in this study 18 cases suffered from postoperative fever higher than 38.5°C (29.5%) including 6 cases (9.8%) of abdominal fluid collection and infection. There were two cases of pancreatic leak with abdominal infection and one case of bile leak with abdominal infection. All six cases with abdominal fluid collection and infection recovered after drainage or antibiotic treatment.

### Is absorbable hernia graft a better alternative to non-absorbable prolene-mesh?

Prolene-mesh with big pores had contractility of around 20% [12]. It was important to maintain the original size of the pancreas after mesh reinforcement otherwise it might lead to pancreatic atrophy or pancreatic duct dilation after surgery.

**Figure 10:**
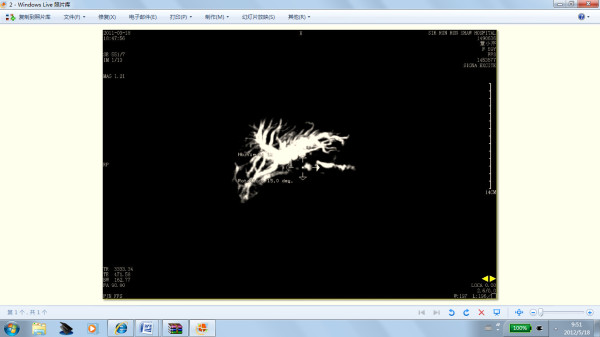
MRI showed pancreatic duct was dilated after surgery during follow-up

Although a stent was inserted into the pancreatic duct, pancreatic atrophy or pancreatic duct dilation may be observed on CT or magnetic resonance imaging (MRI) during the first year of follow-up. This might be attributable to mesh contractility which leads to occlusion of the pancreatic duct. In our study two cases had pancreatic atrophy and pancreatic duct dilation (Figure 10). In a dog experiment, we found that the pancreatic ducts of a few of dogs were dilated or mesh was rejected and dropped into the bowel 3 months after prolene-mesh-reinforced pancreatojejunostomy. However, the pancreatic duct was too fine to insert the pancreatic duct stent in the dog.

It was reported in a human study that end-to-end invaginated pancreaticojejunostomy could cause pancreatic exocrine insufficiency after surgery [13]. Prolene-mesh may make things worse due to its contractility. As far as this was concerned, Biodesign hernia graft (porcine small intestine submucosa, Cook, Limerick, Ireland) was explored from June 2011. Our study demonstrated that a Biodesign hernia graft had no contractility and could grow into autologous tissue 3 to 4 months later. Until November 2011 no pancreatic atrophy or pancreatic duct dilation occurred in the six cases in which Biodesign hernia graft was used. So Biodesign hernia graft might be a better alternative than prolene-mesh.

However, prolene-mesh can promote tissue adherance, which absorbable mesh does not [11]. Further follow-up was required to compare the two types of meshes.

## Conclusions

Mesh-reinforced pancreatojejunostomy and pancreatogastrostomy might be a safer procedure to decrease the incidence of pancreatic leak, especially for tough cases with edematous or fragile pancreatic texture. A prospective randomized trial was required to prove its feasibility in future.

## Abbreviations

CT, Computed tomography; MRI, Magnetic resonance imaging.

## Competing interests

Authors declared there were no competing interests.

## Authors’ contributions

WXF contributed to concept, study design, data analysis, results interpretation and manuscript writing. XY participated in study conceptualization, data collection and drafting the manuscript. ZNY and PJH contributed to conduct of study and data collection. ZW contributed to the conducting of the study, data collection and proof reading. All authors read and approved the final manuscript.

## References

[B1] Buchler MW Friess H Wagner M Kulli C Wagener V Z’Graggen K Pancreatic fistula after pancreatic head resection Br J Surg 2000 87 883 889 10.1046/j.1365-2168.2000.01465.x 10931023

[B2] Bassi C Dervenis C Butturini G Fingerhut A Yeo C Izbicki J Neoptolemos J Sarr M Traverso W Buchler M Postoperative pancreatic fistula: an international study group (ISGPF) definition Surgery 2005 138 8 13 10.1016/j.surg.2005.05.001 16003309

[B3] Hosotani R Doi R Imamura M Duct-to-mucosa pancreaticojejunostomy reduces the risk of pancreatic leakage after pancreatoduodenectomy World J Surg 2002 26 99 104 10.1007/s00268-001-0188-z 11898041

[B4] Bassi C Falconi M Molinari E Mantovani W Butturini G Gumbs AA Salvia R Pederzoli P Duct-to-mucosa versus end-to-side pancreaticojejunostomy reconstruction after pancreaticoduodenectomy: results of a prospective randomized trial Surgery 2003 134 766 771 10.1016/S0039-6060(03)00345-3 14639354

[B5] Stojadinovic A Brooks A Hoos A Jaques DP Conlon KC Brennan MF An evidence-based approach to the surgical management of resectable pancreatic adenocarcinoma J Am Coll Surg 2003 196 954 964 10.1016/S1072-7515(03)00010-3 12788434

[B6] Huang DY Wang XF Zhou W Xin Y Mou YP Cai XJ Polypropylene mesh-reinforced pancreaticojejunostomy for periampullar neoplasm World J Gastroenterol 2007 13 6072 6075 10.3748/wjg.13.6072 18023102 PMC4250893

[B7] Li W Wang XF Xu B Zhu YP Mou YP Polypropylene mesh wrap around reinforced pancreatojejunostomy for soft pancreas Zhonghua Yi Xue Za Zhi 2009 89 1391 1394 19671329

[B8] Peng SY Wang JW Lau YW Cai XJ Mou YP Liu YB Li JT Conventional versus binding pancreaticojejunostomy after pancreaticoduodenectomy: a prospective randomized trial Ann Surg 2007 245 692 698 10.1097/01.sla.0000255588.50964.5d 17457161 PMC1877076

[B9] Peng SY Mou YP Liu YB Su Y Peng CH Cai XJ Wu YL Zhou LH Binding pancreaticojejunostomy: 150 consecutive cases without leakage J Gastrointest Surg 2003 7 898 900 10.1007/s11605-003-0036-6 14592664

[B10] Wang X Zhou W Xin Y Huang D Mou Y Cai X A new technique of polypropylene mesh-reinforced pancreaticojejunostomy Am J Surg 2007 194 413 415 10.1016/j.amjsurg.2006.08.091 17693294

[B11] Satoi S Toyokawa H Yanagimoto H Yamamoto T Hirooka S Yui R Yamaki S Matsui Y Mergental H Kwon AH Reinforcement of pancreticojejunostomy using polyglycolic acid mesh and fibrin glue sealant Pancreas 2011 40 16 20 10.1097/MPA.0b013e3181f82f55 20966808

[B12] Amid PK Classification of biomaterials and their related complications in abdominal wall hernia surgery Hernia 1997 1 15 21 10.1007/BF02426382

[B13] Nordback I Parviainen M Piironen A Räty S Sand J Obstructed pancreaticojejunostomy partly explains exocrine insufficiency after pancreatic head resection Scand J Gastroenterol 2007 42 263 270 10.1080/00365520600849174 17327947

